# Supplementary material for: Identification of photoperiod-induced specific miRNAs in the adrenal glands of Sunite sheep (Ovis aries)
Source: Front Vet Sci. 2022 Jul 22;9:888207. doi: 10.3389/fvets.2022.888207 (PMC9354845; doi:10.3389/fvets.2022.888207)
Supplement: Supplementary file 1 [file Data_Sheet_1.ZIP › Supplementary file/miRDeep2 file.zip!/miRDeep2 file/Novel_Structure/SP42A1/Novel_31.pdf]

Provisional ID : NW_014639514.1_27307
Score total : 123.6
Score for star read(s) : 3.9
Score for read counts : 115.9
Score for mfe : 2.3
Score for randfold : 1.6
Score for cons. seed :
Total read count : 239
Mature read count : 232
Loop read count : 0
Star read count : 7

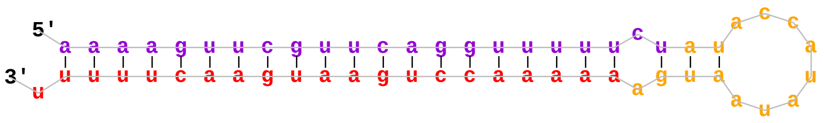

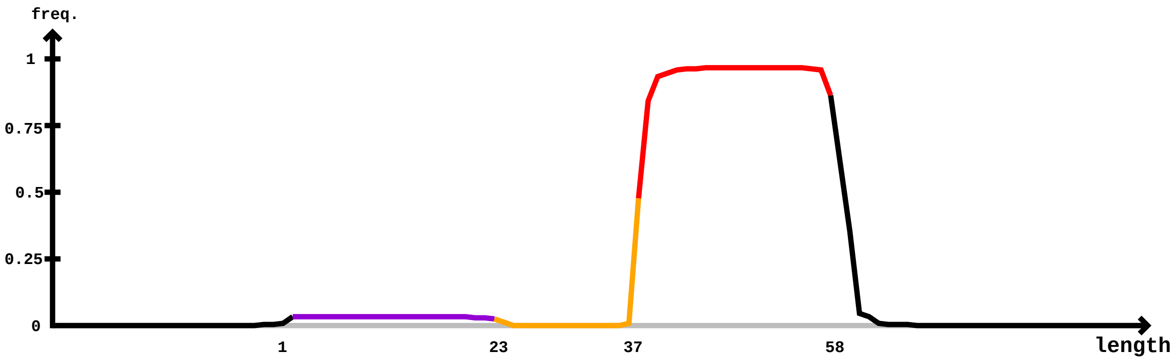

Star Mature

```
5'- uuccauaaauacuggguugggcaaaaaguucguucagguuuuucuauaccauauaaugaaaaaaccugaaugaacuuuuuggccaacccuguauuuuccuugguggcugcaa  -3'  obs
    uuccauaaauacuggguugggcaaaaaguucguucagguuuuucuauaccauauaaugaaaaaaccugaaugaacuuuuuggccaacccuguauuuuccuugguggcugcaa       exp
    ..((((((((((.(((((((.((((((((((((((((((((((.(((........))).)))))))))))))))))))))).))))))).)))))).......))))......   reads  mm  sample
    ....................gcaaaaaguucguucagguuuu......................................................................   1      0   seq
    ......................aaaaaguucguucGgguuuuuc....................................................................   1      1   seq
    .......................aaaaguucguucGgguuuuucu...................................................................   6      1   seq
    ..........................................................Gaaaaaccugaaugaacuuu..................................   1      1   seq
    ..........................................................Gaaaaaccugaaugaacuuuuu................................   1      1   seq
    ...........................................................aaaaaccugaaugaacCu...................................   1      1   seq
    ...........................................................aaaaaUcugaaugaacuuuu.................................   17     1   seq
    ...........................................................aaaaaccugaaCgaacuuuuu................................   20     1   seq
    ...........................................................aaaaaccugaaugaacuuuuC................................   2      1   seq
    ...........................................................aaaaaccAgaaugaacuuuuu................................   2      1   seq
    ...........................................................aaaaaccugaaugaacCuuuu................................   2      1   seq
    ...........................................................aaaaaUcugaaugaacuuuuu................................   42     1   seq
    ...........................................................aaaaaccugaaCgaacuuuuug...............................   11     1   seq
    ...........................................................aaaGaccugaaugaacuuuuug...............................   1      1   seq
    ...........................................................aaaaaccugaaugaacuuuCug...............................   1      1   seq
    ...........................................................aaaaaccugaaGgaacuuuuug...............................   12     1   seq
    ...........................................................aaaaaccugaaugaacuuuuugg..............................   2      0   seq
    ............................................................aaaaccAgaaugaacuuu..................................   2      1   seq
    ............................................................aaaaccugaaugaacuuu..................................   4      0   seq
    ............................................................aaaaccugaaugaacCuuuu................................   1      1   seq
    ............................................................aaaaAcugaaugaacuuuuu................................   2      1   seq
    ............................................................aaaaccugaaCgaacuuuuu................................   1      1   seq
    ............................................................aaaaccugaGugaacuuuuu................................   1      1   seq
    ............................................................aaaaccAgaaugaacuuuuu................................   4      1   seq
    ............................................................aaaaccugaaugaacuuuuG................................   1      1   seq
    ............................................................Gaaaccugaaugaacuuuuug...............................   2      1   seq
    ............................................................aaaaccugaaugaacuuuuug...............................   10     0   seq
    ............................................................aaaaccAgaaugaacuuuuug...............................   2      1   seq
    ............................................................aaaaccugaaugaacuuuuuU...............................   3      1   seq
    ............................................................aaaaccugaaGgaacuuuuug...............................   1      1   seq
    ............................................................aaaaccugaaugaacCuuuug...............................   1      1   seq
    ............................................................aaaaccugaaugaacuuuuugA..............................   13     1   seq
    ............................................................aaaaccugaGugaacuuuuugg..............................   1      1   seq
```

```
                       Star                                Mature
uuccauaaauacuggguugggcaaaaaguucguucagguuuuucuauaccauauaaugaaaaaaccugaaugaacuuuuuggccaacccuguauuuuccuugguggcugcaa
............................................................aaaaccugaaugaacuuuuuU...............................   4    1    seq
............................................................aaaaccugaaugaacuuuuugg..............................   32   0    seq
............................................................aaaaccugaaugGacuuuuugg..............................   1    1    seq
............................................................aaaaccugaaugaacuuuuuggG.............................   2    1    seq
.............................................................aaaccugaaugaacuuuuugA..............................   1    1    seq
.............................................................aaaccCgaaugaacuuuuugg..............................   17   1    seq
.............................................................aaaccugaaugaacuuuuugg..............................   2    0    seq
.............................................................aaaccugaaugaacuuuuugU..............................   1    1    seq
.............................................................aaaccugaGugaacuuuuuggc.............................   1    1    seq
..............................................................aaccugaaugaacuuuuuggcc............................   1    0    seq
..............................................................aaccugaaugaacuuuuuggcU............................   2    1    seq
...............................................................accCgaaugaacuuuuuggcc............................   1    1    seq
...............................................................accugaaugaacuuuuugAcc............................   1    1    seq
...............................................................accugaaugaacuuuuuggcca...........................   1    0    seq
................................................................ccugaaugaacuuuuuggcc............................   1    0    seq
..................................................................ugaaugaacuuuuuggccCacc........................   1    1    seq
```
